# Exploring the association between serum phosphate levels and mortality in patients hospitalized with infectious diseases: a nationwide study

**DOI:** 10.3389/fmed.2024.1362106

**Published:** 2024-03-25

**Authors:** Amit Frenkel, Adi Shiloh, Victoria Vinokur, Matthew Boyko, Yair Binyamin, Jacob Dreiher

**Affiliations:** ^1^General Intensive Care Department, Soroka University Medical Center, and The Faculty of Health Sciences, Ben-Gurion University of the Negev, Beer-Sheva, Israel; ^2^Clinical Research Center, Soroka University Medical Center, The Faculty of Health Sciences, Ben-Gurion University of the Negev, Beer-Sheva, Israel; ^3^Division of Anesthesiology and Critical Care, Soroka University Medical Center, The Faculty of Health Sciences, Ben-Gurion University of the Negev, Beer-Sheva, Israel; ^4^Hospital Administration, Soroka University Medical Center, The Faculty of Health Sciences, Ben-Gurion University of the Negev, Beer-Sheva, Israel

**Keywords:** phosphate, mortality, infection, target organ damage, length of stay

## Abstract

**Objective:**

The purpose of this study was to examine associations of serum phosphate levels with mortality, target organ damage and length of hospital stay in adults with infectious diseases hospitalized outside of the intensive care unit.

**Methods:**

This nationwide retrospective cohort study comprised patients admitted with infections, to medical and surgical departments in eight tertiary hospitals during 2001–2020. The main exposure variable was the first serum phosphate levels at admission (up to 1 week). The analysis included multivariable logistic regression models and quantile regression.

**Results:**

Of 126,088 patients (49% males, mean age: 69.3 years), 24,809 (19.7%) had decreased phosphate levels, 92,730 (73.5%) normal phosphate levels, and 8,549 (6.8%) elevated phosphate levels on admission. Overall- and in-hospital mortality rates were highest among those with hyperphosphatemia (74.5 and 16.4%, respectively), followed by those with normophosphatemia (57.0 and 6.6%), and lastly the hypophosphatemia group (48.7 and 5.6%); *p* < 0.001 for all. After adjusting for confounders, the lowest predicted mortality rate was observed in the normophosphatemia group. In the multivariable model, hyperphosphatemia conferred a higher probability of target organ damage (OR [95% CI]: 2.43 [2.06–2.86]), while moderate hypophosphatemia conferred a lower probability (OR [95% CI]: 0.73 [0.65–0.82]), compared to normal phosphate levels and extreme hypophosphatemia showed a non-significant association (OR [95% CI]: 0.87 [0.57–1.28]). The associations were independent of renal failure. In a multivariable model, hyperphosphatemia was associated with a slight increase of 0.33 days in length of stay compared to normal phosphate levels.

**Conclusion:**

A J-shaped relation was found between phosphate levels and prognosis in patients hospitalized with infectious diseases, regardless of their renal function.

## Introduction

Infectious diseases continue to pose a significant global health burden, causing substantial morbidity and mortality worldwide. While considerable efforts have been invested to improve treatment strategies and reduce the adverse outcomes associated with infections, identifying additional prognostic markers that can help predict patient outcomes remains a vital area of research. One such potential marker of interest is serum phosphate level, which has been implicated in various physiological processes and disease states.

Phosphate, an indispensable mineral vital for cellular energy metabolism, bone mineralization and a myriad of enzymatic reactions, plays a fundamental role in maintaining homeostasis throughout the human body (1). Perturbations in phosphate levels have been linked to a wide range of pathological conditions, encompassing renal dysfunction, cardiovascular disease and metabolic disorders. Furthermore, within the cellular landscape, phosphate assumes a central role as a valuable “currency.” The paramount energy carrier, adenosine triphosphate (ATP), relies on phosphate bonds to liberate and transfer energy during cellular metabolic processes (2). Additionally, phosphate actively engages in cellular signalling pathways, mediating crucial phosphorylation events that govern enzyme activity and gene expression, thereby influencing diverse cellular functions such as growth, proliferation and differentiation (3).

In an interesting study published in 2022, Yang et al. examined the association between phosphate levels and mortality in critically-ill patients admitted to the intensive care unit (ICU) for various reasons (4). Among 24,289 patients, elevated phosphate levels were independently associated with an increased risk of ICU mortality, hazard ratio 1.06 (95% confidence interval (CI): 1.02–1.09, *p* < 0.001). Two other studies (5, 6), both published in 2023, indicated similar results, but focused specifically on patients with sepsis. Among 1,855 patients with sepsis, Black et al. (5) reported higher mortality among those with phosphate levels in the highest quartile (>4.0 mg/dL) than in the three lower quartiles. Among 9,691 patients with sepsis, Xu et al. (6), observed higher 28-day mortality among those with hyperphosphatemia within 2 days of ICU admission. Importantly, the above studies included only patients with sepsis who were hospitalized in critical care units.

Though most patients with infectious diseases are hospitalized in medical and surgical wards, rather than the ICU, only scarce research has investigated the relation between phosphate levels on admission and mortality rates. Consequently, the objective of this nationwide study was to examine a possible association between serum phosphate levels and mortality rates in patients with infectious diseases hospitalized outside the ICU.

## Materials and methods

### Study population

We conducted a multicenter population-based retrospective cohort study of patients hospitalized at any of eight academic medical centers owned by Clalit Health Services (CHS). CHS is the largest healthcare provider in Israel, with over 4.6 million enrolees. The current cohort was previously described (7). All CHS members hospitalized between December 2001 and October 2020 with a primary diagnosis of infectious disease based on the International Classification of Diseases, 9th revision clinical modification (ICD-9-CM) diagnoses codes were included. To avoid including patients with nosocomial infections, which may possess unique and distinguished characteristics, only patients with infections documented within the first day of hospital admission were included. Hospitalization length of stay was restricted to 3–30 days. This limited the cohort to patients with a substantial illness that required hospitalization, whether acute or sub-acute. According to the methodology developed by Martin et al. (8), sources of infection were categorized according to the ICD-9-CM codes: pulmonary, cardiovascular, skin and soft tissue, urinary, nervous system, gastrointestinal and peritoneum, bone and joints, and bacteremia of unspecified site.

### Data sources and clinical definitions

Demographics (age, sex), hospitalization details (length of stay, ICU transfers) and laboratory blood results from the index hospitalization were collected from the hospital records. Data were retrieved of underlying medical conditions according to the chronic disease registries of CHS, and of chronic medications purchased 3 months prior to hospitalization based on the Anatomical Therapeutic Chemical (ATC) coding of the World Health Organization’s classification system. Target organ damage was defined using the methodology described by Martin et al. (8), based on ICD-9-CM codes for acute organ system failure (respiratory: 518.5–518.53, 518.81, 518.82, 518.84, 518.85, 786.09, 799.1, Z79.7, Z96.70–Z96.72; cardiovascular: 427.5, 458.0, 458.8, 458.9, 785.5, 785.50–785.52, 785.59, 785.591, 796.3; renal: 580, 580.0, 580.4, 580.81, 580.89, 580.9, 584, 584.5–584.9, 585, Z39.95; liver: 570, 572.2, 573.3; hematologic: 286.6, 286.9, 287.30–287.32, 287.4, 287.49, 287.5; metabolic: 276.2; neurologic: 293, 293.0, 293.1, 293.9, 348.1, 348.3, 348.31, 348.39, 780.01, 780.09, Z89.14; nonspecific: 995.92). Renal failure was defined as a composite exposure variable based on the presence of at least one of the following: a diagnosis of acute kidney injury, chronic renal disease or renal failure; or estimated glomerular filtration rate (eGFR) < 60 mL/min (9).

The main exposure variable was serum levels of phosphate on admission (up to 1 week). The first phosphate levels were used to examine associations with the outcome variables. Phosphate levels were categorized as extreme hypophosphatemia (<1.5 mg/dL), moderate hypophosphatemia (1.5–2.5 mg/dL), normophosphatemia (2.5–4.5 mg/dL) or hyperphosphatemia (> 4.5 mg/dL) (10).

Outcome variables included one-year and 30-day mortality, target organ damage during hospitalization (one or more), and median hospitalization length of stay (considering only patients who survived the hospitalization).

Data were extracted from the CHS database using a data-sharing platform powered by MDClone. The study was conducted according to the guidelines of the Declaration of Helsinki, and approved by the Institutional Review Board of Soroka Medical Center (protocol number 0108-16-SOR). No informed consent was needed.

### Statistical analysis

When appropriate, univariate comparisons were made using the Chi-square test or Fisher’s exact test for categorical variables, and using the analysis of variance (one-way ANOVA) or Kruskal-Wallis tests for quantitative or ordinal variables. Survival curves were generated using the Kaplan–Meier method. Multivariable logistic regression was used to model the factors associated with 30-day mortality and target organ damage, and quantile regression was used for assessing factors associated with the median hospitalization length of stay. Multivariable cubic spline regression was used to estimate the nonlinear relation between phosphate level and 30-day mortality, with three knots placed at phosphate upper and lower levels (1.5, 2.5 and 4.5 mg/dL). Variables were evaluated as potential confounders according to the results of the univariate analysis (*p* < 0.1) or their clinical importance. For the point estimates of odds ratios (ORs) or coefficients in the quantile regression, 95% CIs were calculated, and rounded outwards. Due to the large sample size, a two-sided *p*-value of <=0.01 was considered statistically significant. IBM SPSS software, version 26.0, and R Statistical Software (version 4.2.1; R Core Team 2022) was used for the statistical analysis.

## Results

### Patient characteristics

A total of 126,088 patients were included in this study (49% males, mean age: 69.3 years). Of them, 24,809 (19.7%) had low phosphate levels, 92,730 (73.5%) had phosphate levels within the normal range, and 8,549 (6.8%) had high phosphate levels on admission. Phosphate levels tended to be higher among older patients and those of female sex. The prevalence of comorbidities such as diabetes mellitus, ischemic heart disease, past stroke, malignancies, liver cirrhosis, congestive heart failure, chronic renal failure, hypertension and atrial fibrillation/flutter were higher in the hyperphosphatemia than the normophosphatemia and hypophosphatemia groups (Table 1).

**Table 1:** Demographic characteristics and hospitalization details of the cohort according to phosphate level on admission (up to 1 week).

	Hypophosphatemia *p* ≤ 2.5 *n* = 24,809	Normophosphatemia 2.5 < *p* ≤ 4.5 *n* = 92,730	Hyperphosphatemia 4.5 < *p n* = 8,549	*p*-value
Age, mean ± SD	67.6 ± 18.6	69.5 ± 18.7	72.2 ± 17.1	<0.001
Male sex, No. (%)	13,393 (54.0)	44,443 (47.9)	3,970 (46.4)	<0.001
Diabetes mellitus, No. (%)	7,978 (32.2)	34,045 (36.7)	4,143 (48.5)	<0.001
Ischemic heart disease, No. (%)	7,001 (28.2)	30,769 (33.2)	3,744 (43.8)	<0.001
Hematologic malignancy, No. (%)	1,030 (4.2)	4,020 (4.3)	471 (5.5)	<0.001
Solid malignancy, No. (%)	12,975 (52.3)	51,625 (55.7)	5,221 (61.1)	<0.001
Liver cirrhosis, No. (%)	424 (1.7)	1,395 (1.5)	201 (2.4)	<0.001
Congestive heart failure, No. (%)	3,097 (12.5)	19,050 (20.5)	3,196 (37.4)	<0.001
Chronic renal failure, No. (%)	4,122 (16.6)	20,674 (22.3)	4,079 (47.7)	<0.001
COPD, No. (%)	3,915 (15.8)	21,157 (22.8)	2,755 (32.2)	<0.001
Hypertension, No. (%)	14,367 (57.9)	59,428 (64.1)	6,474 (75.7)	<0.001
Creatinine (max), mg/dL, median (IQR)	1 (0.8–1.3)	1 (0.8–1.4)	1.6 (1–2.5)	<0.001
eGFR <60 mL/min, No. (%)	7,538 (30.7)	32,107 (35.2)	4,776 (73.4)	<0.001
Overall mortality, No. (%)	12,094 (48.7)	52,876 (57)	6,370 (74.5)	<0.001
In-hospital mortality, No. (%)	683 (5.6)	3,478 (6.6)	1,042 (16.4)	<0.001
30-day mortality, No. (%)	755 (6.2)	3,672 (6.9)	627 (9.8)	<0.001
ICU transfer, No. (%)	525 (2.1)	1,386 (1.5)	359 (4.2)	<0.001
Hospitalization length of stay, median (IQR)	5 (4–7)	5 (3–7)	6 (4–9)	<0.001
Any target organ damage, No. (%)	2,723 (11.0)	11,345 (12.2)	2,627 (30.7)	<0.001
Respiratory	565 (2.3)	2,818 (3)	580 (6.8)	<0.001
Vascular	218 (0.9)	744 (0.8)	214 (2.5)	<0.001
Renal	1,553 (6.3)	6,761 (7.3)	1946 (22.8)	<0.001
Liver	98 (0.4)	443 (0.5)	66 (0.8)	<0.001
Hematologic	492 (2.0)	1,209 (1.3)	172 (2.0)	<0.001
Metabolic	66 (0.3)	342 (0.4)	128 (1.5)	<0.001
CNS	23 (0.1)	113 (0.1)	2 (0)	0.021
Nonspecific	336 (1.4)	1,084 (1.2)	368 (4.3)	<0.001

SD, standard deviation; COPD, chronic obstructive pulmonary disease; ICU, intensive care unit; IQR, interquartile range; CNS, central nervous system; eGFR, estimated glomerular filtration rate.

**Figure 1:**
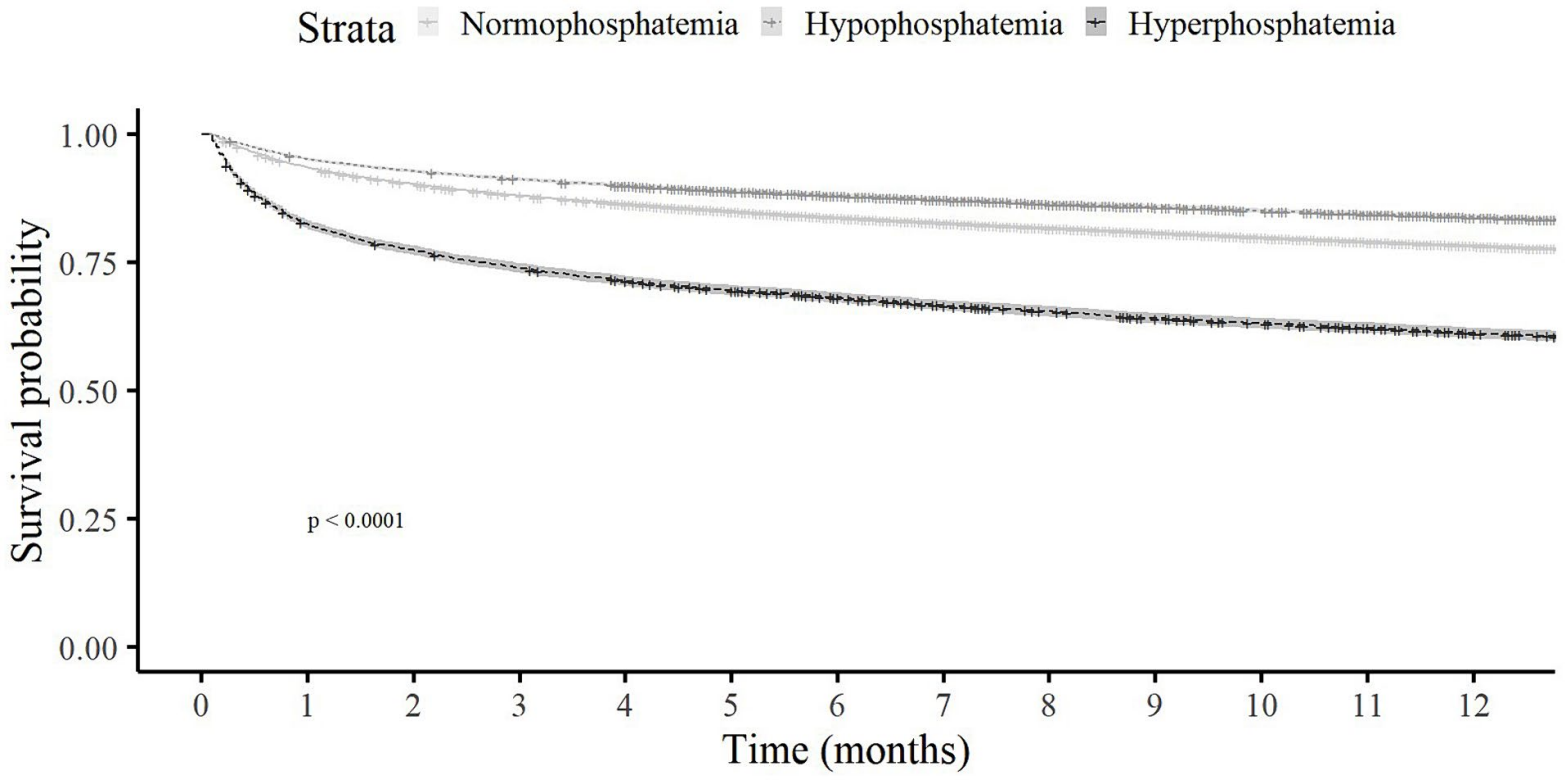
Kaplan–Meier survival duration estimates 1 year following hospitalization.

Overall and in-hospital mortality rates were highest among those with hyperphosphatemia (74.5 and 16.4%, respectively), followed by those with normophosphatemia (57 and 6.6%, respectively), and lastly the hypophosphatemia group (48.7 and 5.6%, respectively); *p* < 0.001 for all. In a landmark analysis of patients who survived to hospital discharge, among those with hyperphosphatemia compared to normal and low phosphate, the cumulative survival rate at 1 year following hospitalization was lower (Figure 1). The frequency of transfers to the ICU was also higher for the hyperphosphatemia than the normophosphatemia and hypophosphatemia groups. Target organ damage was more common in the hyperphosphatemia than the normophosphatemia and hypophosphatemia groups (30.7, 12.2 and 11%, respectively). Renal damage was the most frequent target organ damage in all the groups.

### Clinical outcomes

Table 2 presents multivariable logistic regression models of factors associated with 30-day mortality. For both patients with and without renal failure, age, target organ damage and albumin level < 2.5 g/dL were significant. Diabetes diagnosis was associated with 30-day mortality only for patients without renal failure. Compared to normal phosphate levels, high phosphate levels were associated with higher mortality, while moderately low phosphate levels were associated with lower mortality. These relations were evident among patients with renal failure (OR [95% CI] for extreme hypophosphatemia, moderate hypophosphatemia and hyperphosphatemia: 0.72 [0.44–1.12], 0.57 [0.50–0.65] and 2.15 [1.95–2.37], respectively) and without renal failure (OR [95% CI] 0.99 [0.67–1.52], 0.74 [0.64–0.84] and 2.18 [1.79–2.63], respectively).

**Table 2:** Factors associated with 30-day mortality (multivariable logistic regression).

	All patients		Patients with renal failure		Patients without renal failure	
	OR (95% CI)	*p*-value	OR (95% CI)	*p*-value	OR (95% CI)	*p*-value
Age (for every additional 5 years)	1.32 (1.30–1.33)	<0.001	1.30 (1.28–1.32)	<0.001	1.34 (1.32–1.36)	<0.001
Male sex	1.06 (1.01–1.12)	0.013	1.05 (0.99–1.12)	0.086	1.07 (0.99–1.16)	0.091
Diabetes mellitus	1.05 (1.00–1.10)	0.042	1.03 (0.97–1.09)	0.4	1.09 (1.00–1.18)	0.042
Potassium <3.5 mEq/L	1.36 (1.24–1.49)	<0.001	1.25 (1.11–1.42)	<0.001	1.48 (1.29–1.68)	<0.001
No target damage (reference)	1.00		1.00		1.00	
Target organ damage to 1 system	1.73 (1.64–1.85)	<0.001	1.54 (1.44–1.65)	<0.001	2.77 (2.44–3.13)	<0.001
Target organ damage to 2 systems or more	3.83 (3.36–4.36)	<0.001	3.60 (3.14–4.12)	<0.001	4.73 (3.10–7.09)	<0.001
Normophosphatemia (reference)	1.00		1.00		1.00	
Extreme hypophosphatemia	0.87 (0.57–1.28)	0.5	0.72 (0.44–1.12)	0.3	0.99 (0.61–1.52)	>0.9
Moderate Hypophosphatemia	0.73 (0.65–0.82)	<0.001	0.57 (0.50–0.65)	<0.001	0.74 (0.64–0.84)	<0.001
Hyperphosphatemia	2.43 (2.06–2.86)	<0.001	2.15 (1.95–2.37)	<0.001	2.18 (1.79–2.63)	<0.001
Albumin <2.5 g/dl	7.98 (7.50–8.49)	<0.001	7.20 (6.65–7.78)	<0.001	9.43 (8.53–10.4)	<0.001
Albumin <2.5*Normophosphatemia (reference)	1.00		1.00		1.00	
Albumin <2.5*Extreme hypophosphatemia	1.09 (0.70–1.73)	0.7	1.39 (0.74–2.66)	0.3	0.77 (0.40–1.48)	0.4
Albumin <2.5*Moderate Hypophosphatemia	1.26 (1.09–1.46)	0.002	1.26 (1.03–1.54)	0.027	1.15 (0.93–1.42)	0.2
Albumin <2.5*Hyperphosphatemia	0.77 (0.66–0.89)	<0.001	0.78 (0.66–0.92)	0.003	1.11 (0.76–1.62)	0.6
Renal failure	1.13 (1.07–1.21)	<0.001				
Renal failure*Normophosphatemia (reference)	1.00					
Renal failure*Extreme hypophosphatemia	0.91 (0.58–1.44)	0.7				
Renal failure*Moderate Hypophosphatemia	0.77 (0.67–0.89)	<0.001				
Renal failure*Hyperphosphatemia	0.88 (0.74–1.05)	0.2				

OR, odds ratio; CI, confidence interval. Normophosphatemia – phosphate 2.5–4.5 mg/dL; Extreme hypophosphatemia < 1.5 mg/dL; Moderate Hypophosphatemia – phosphate 1.5–2.5 mg/dL; Hyperphosphatemia – phosphate > 4.5 mg/dL.

**Figure 2:**
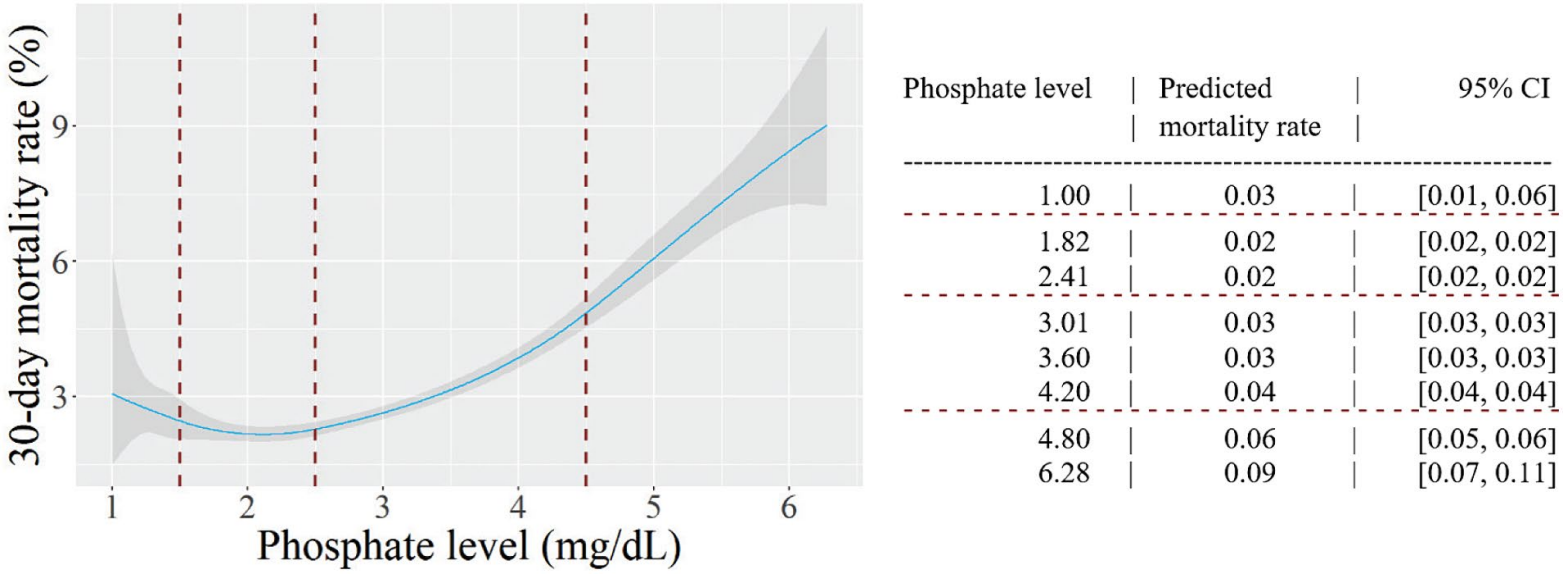
The relation between phosphate level and 30-day mortality. Predicted probabilities and 95% confidence intervals (CI) are shown, based on a multivariable cubic spline regression model adjusted for age, sex, target organ damage, diabetes mellitus, serum albumin level < 2.5 g/dl, serum potassium level < 3.5 mEq/L and renal failure. Knots were placed at phosphate levels of 1.5, 2.5 and 4.5 mg/dL (dashed lines).

Figure 2 presents the results of a cubic spline regression model that examined the nonlinear relation between phosphate level and 30-day mortality. After adjusting for confounders, the results showed a J-shaped curve. Accordingly, the lowest predicted mortality was observed in the normophosphatemia group, and both high and low phosphate levels were associated with increased mortality rates. Specifically, focusing on low phosphate levels, the predicted mortality rate increased from 2 to 3% when the phosphate level decreased to 1.00 mg/dL. In contrast, in the high phosphate group, the predicted mortality rate increased substantially to 10% at a phosphate level of 6.28 mg/dL.

In a multivariable model, age, sex, diabetes diagnosis, low albumin level and renal failure were associated with target organ damage (Table 3). Compared to normophosphatemia, hyperphosphatemia was associated with a higher probability of target organ damage (OR [95% CI]: 2.00 [1.89–2.11]), while hypophosphatemia was associated with a lower probability (OR [95% CI]: 0.92 [0.88–0.97]). The associations were consistent for patients with and without renal failure, and there was no interaction between phosphate level and renal failure.

**Table 3:** Factors associated with target organ damage (multivariable logistic regression).

	OR (95% CI)	*p*-value
Normophosphatemia (reference)	1.00	
Extreme hypophosphatemia	1.22 (1.04–1.42)	0.013
Moderate hypophosphatemia	0.91 (0.87–0.96)	<0.001
Hyperphosphatemia	2.98 (1.87–2.10)	<0.001
Age (for every additional 5 years)	0.99 (0.98–1.00)	<0.001
Male sex	1.23 (1.19–1.28)	<0.001
Diabetes mellitus	1.05 (1.01–1.09)	0.003
Potassium <3.5 mEq/L	0.89 (0.82–0.97)	0.007
Albumin <2.5 g/dl	2.43 (2.32–2.55)	<0.001
Renal failure	5.54 (5.29–5.80)	<0.001

OR, odds ratio; CI, confidence interval. Normophosphatemia – phosphate 2.5–4.5 mg/dL; Extreme hypophosphatemia < 1.5 mg/dL; Moderate Hypophosphatemia – phosphate 1.5–2.5 mg/dL; Hyperphosphatemia – phosphate > 4.5 mg/dL.

In a sensitivity analysis, we examined the association between phosphate levels and mortality according to the source of infection. Results were similar for 54,343 patients with a pulmonary source of infection (for extreme hypophosphatemia, OR [95% CI]: 1.09 [0.74–1.57]; for moderate hypophosphatemia, OR [95% CI]: 0.75 [0.67–0.87] and for hyperphosphatemia OR [95% CI]: 1.92 [1.72–2.13]) and for 28,496 patients with a renal source of infection (for extreme hypophosphatemia, OR [95% CI]: 0.83 [0.39–1.54]; for moderate hypophosphatemia, OR [95% CI]: 0.60 [0.49–0.72] and for hyperphosphatemia OR [95% CI]: 2.35 [1.1.92–2.86]).

## Discussion

In this large study of patients with infections disease hospitalized outside of the ICU, hyperphosphatemia on admission was associated with short- and long-term mortality, target organ damage and longer hospital stay. To a lesser extent, hypophosphatemia was associated with short- and long-term mortality and target organ damage. A multivariate analysis showed a J-shaped association between phosphate levels and mortality.

The findings of the current study, which comprised hospitalized patients with infectious diseases, are in line with the findings of a large study that investigated the association between phosphate levels and mortality (11). That study included 42,336 patients admitted to Mayo Clinic in Rochester over a four-year period, for a wide variety of diseases. Their results, like ours, revealed a J-shaped curve, indicating associations of both low (<3.1 mg/dL) and high (>4.2 mg/dL) serum phosphate levels with higher in-hospital mortality. Even after adjusting for potential confounding factors, both low and high serum phosphate levels remained significantly associated with in-hospital mortality. However, as highlighted earlier, while that study conducted several subgroup analyses, it did not specifically analyze patients with infectious diseases.

The main clinical question that arises from our research is the mechanism linking phosphate levels with the infectious process and prognosis. Stemming from this is the question as to whether phosphate levels are only a prognostic marker or whether correcting them may affect prognosis. Three important elements constitute possible links between hypophosphatemia and infection. First, hypophosphatemia results from acute respiratory alkalosis, a clear sign of the onset of sepsis. In healthy persons, hyperventilation (to partial pressure of carbon dioxide <20 mmHg) can lower serum phosphate concentrations to below 1 mg/dL (0.32 mmol/L) (12). This is probably the most common cause of marked hypophosphatemia in hospitalized patients (13). Second, patients with acute infection tend to develop hyperglycemia and increased insulin secretion. The underlying reasons are elevated cortisol levels and increased secretion of catecholamines and glucagon, as well as increased gluconeogenesis and glycogenolysis (14). Insulin secretion in itself is directly related to hypophosphatemia (15). Lastly, severe infectious disease may lead to decreased intestinal absorption (16), thus resulting in hypophosphatemia. Similarly, it is worth highlighting the two main factors associated with an infectious process and causing hyperphosphatemia. The first is the decreased renal clearance seen in acute kidney injury, which often accompanies acute infections in hospitalized patients (17). The second is the cellular shift causing hyperphosphatemia, which results from acute lactic acidosis (18). This classic phenomenon accompanies severe infectious diseases, especially those that include a decrease in cell perfusion. We suspect that the non-symmetrical J-shaped curve of the association between phosphate levels and mortality can be explained by the more clinically significant causes of hyperphosphatemia that relate to poor prognosis, rather than the more benign causes of hypophosphatemia.

Regarding the well-known association between hyperphosphatemia and renal failure mentioned above, we emphasize that we analysed specifically the association between hyperphosphatemia and mortality, while statistically neutralizing the accompanying renal failure. Indeed, this study identified phosphate as a significant prognostic marker regardless of renal failure. This supports the possibility that hyperphosphatemia is more likely related to target organ damage and tissue degradation than to renal failure itself.

Our study has a number of limitations. First, the phosphate levels were measured over a period of up to 1 week from hospital admission, and not at the same point in time for all the patients included in the study. Second, an unknown proportion of patients with infection actually qualified for the diagnostic criteria of sepsis, and it is not always feasible to discern these from those who did not qualify. Another shortcoming of the study is the inability to distinguish patients with acute and chronic renal failure. Finally, the study relies on administrative data, which is usually not intended for clinical research. However, the methodology is well-established, both in our previous studies of the same cohort (19, 20) and in a leading international study in this field, conducted by Martin et al. (8) and published in 2003.

A key strength of the present study is the size of the population and its scope. Our cohort contained data collected over about 20 years, from eight hospitals. For both before and after hospital admission, the study utilized the extensive uniform database of CHS, with its rich demographic and clinical data. These included confounders such as underlying comorbidities and chronic medications, thus enabling robust multivariate analysis.

To conclude, the present study found a J-shaped relation between phosphate levels and prognosis in patients hospitalized with infectious diseases, regardless of their renal function. Future clinical studies regarding the pathophysiology and therapeutic implications of these findings should explore the utility of correcting phosphate levels and clarify whether phosphate abnormalities are more than just a marker of target organ damage.

## Data availability statement

The original contributions presented in the study are included in the article/supplementary material, further inquiries can be directed to the corresponding author.

## Ethics statement

The studies involving humans were approved by Soroka medical center ethics committee. The studies were conducted in accordance with the local legislation and institutional requirements. Written informed consent for participation was not required from the participants or the participants’ legal guardians/next of kin in accordance with the national legislation and institutional requirements.

## Author contributions

AF: Supervision, Writing – original draft. AS: Data curation, Methodology, Software, Writing – original draft. VV: Validation, Writing – review & editing. MB: Formal analysis, Project administration, Writing – review & editing. YB: Formal analysis, Writing – review & editing. JD: Supervision, Methodology, Writing – original draft.
